# The effect of buffer strip width and selective logging on streamside plant communities

**DOI:** 10.1186/s12898-019-0225-0

**Published:** 2019-02-09

**Authors:** A. Oldén, V. A. O. Selonen, E. Lehkonen, J. S. Kotiaho

**Affiliations:** 1 Department of Biological and Environmental Science, University of Jyväskylä, P.O. Box 35, FI-40014 Jyväskylä, Finland; 2 School of Resource Wisdom, University of Jyväskylä, P.O. Box 35, FI-40014 Jyväskylä, Finland; 3 Remsoil Oy, Santastentie 197, 38950 Honkajoki, Finland; 4 Tampere, Finland

**Keywords:** Biodiversity, Conservation, Forest management, Mosses, Selective logging, Vascular plants, Woodland key habitats

## Abstract

**Background:**

Riparian forests surrounding streams host high biodiversity values, but are threatened by clear-cut logging. Narrow buffer strips of about 15 m are commonly left between the stream and the clear-cut, but studies suggest that the buffer width should be at least 30 m to protect riparian plant communities. Moreover, selective logging is often allowed on the buffer strips in order to increase economic gain. We used an experiment of 43 riparian sites where buffer strip width and selective logging within the strip were manipulated and supplemented with unlogged control sites. We report the short-term changes in the community composition of vascular plants and mosses near the stream (0–15 m distance).

**Results:**

15-meter buffers are not enough to protect the vascular plant communities from changes caused by a clear-cut irrespective of the selective logging on the buffer strip. For moss communities 15-m buffers were not enough if they were selectively logged. Relative to the control sites, we observed no significant changes in community composition of vascular plants or mosses in the sites with 30-m buffer strips, whether selectively logged or not.

**Conclusions:**

We conclude that buffer strips of 15 m are not sufficient to protect streamside plant communities even in the short term, but that buffers of 30 m should be left on both sides of the stream. Selective logging appears not to have effects on buffers that are at least 30 m wide. Thus, it may be more reasonable to increase buffer width and to allow selective logging on the wider buffer in order to compensate for the economic losses than to leave all trees on a narrow and ecologically insufficient buffer.

**Electronic supplementary material:**

The online version of this article (10.1186/s12898-019-0225-0) contains supplementary material, which is available to authorized users.

## Background

Demands for ecologically sustainable forestry are increasing worldwide, but the economical cost-effectiveness of forest management is required simultaneously. In Fennoscandia, the dominant forest management method is still periodic cover silviculture with final felling by clear-cutting [1, 2]. Clear-cut management decreases forest biodiversity and ecological values [1–3]. In the mid-1990’s the Fennoscandian and Baltic countries adopted new forestry measures that aim to integrate biodiversity concerns into production forestry [4, 5]. Among these measures is the conservation of woodland key habitats (WKHs) that are small habitat patches with high conservation value. Setting aside and protecting WKHs is assumed to be a cost-effective tool in the conservation of biodiversity in managed forests [6, 7]. However, since WKHs are small patches [7, 8], they are very vulnerable to negative edge effects from the surrounding clear-cuts [9–12].

In the Scandinavian countries, streamside riparian forests are a common type of WKHs [7, 8]. They are characterized by high soil water level, flooding, moist and cool microclimatic conditions, and plant communities dependent on those conditions [12–15]. To protect the stream as well as the riparian microclimate and species communities from the effects of clear-cutting, unlogged buffer strips are required between the stream and the clear-cut area [11–13, 16]. Forested buffer strips can emulate the natural disturbance dynamics of such moist habitats that were rarely affected by large-scale stand-replacing disturbances, e.g. forest fires, but were disturbed by gap dynamics [17]. Such disturbances created corridors and networks of forest that was characterized by a relatively moist and stable microclimate and a continuous supply of dead wood [17].

Buffer strips of 10 m (on both sides of the stream) conserve more species than clear-cut streamsides, but the most sensitive species decline [11] and community composition changes [16]. Studies have suggested that to conserve the plant species of the riparian habitat, it is necessary to leave buffer strips of 30 m [16] or even 45 m [12]. Buffers of 30 m are also sufficient for the protection of stream water quality and aquatic species, but wider buffers of at least 100 m are required for protecting terrestrial riparian wildlife [18, 19]. To limit the economic losses from retaining the buffers, selective logging can be allowed on the buffer strip [20]. Selective logging increases shrub and sapling regeneration on the buffer [21] and has small impacts on bird densities [22]. Carlson et al. [23] found that selective logging on the buffer increases the density of stream macroinvertebrates, but Kreutzweiser et al. [24] reported that it may not have much of an impact beyond the impact of the upland clear-cutting. However, it is uncertain whether riparian buffer strips can tolerate selective logging without changes in microclimate and species composition.

In Finland, those streamside habitats that are in natural or semi-natural condition are protected by the Forest Act [25]. The act forbids altering their characteristic features, which are the special growing conditions and the microclimate that result from the proximity of water and the tree and shrub layers [25]. Within the habitat it is forbidden to perform detrimental actions, such as regeneration felling, forest road construction or cleaning the stream channel [25]. However, it is allowed to undertake management actions that preserve the characteristic features, such as cautious selective logging by picking individual trees, as long as the stand structure and water economy are maintained in their natural or nearly natural state [25]. The act protects the “immediate surroundings” of the stream, but does not state how far this area reaches from the stream, and does not describe how the special growing conditions and microclimate are to be preserved if the upland forest is clear-cut. In practice, forested buffer strips are left between the stream and the clear-cut area, with the aim of protecting also the habitats and species within the buffer strip. Interestingly, the forest authorities call the whole buffer strip the “immediate surrounding”, i.e. the habitat protected by the act [26], although it is obvious that the microclimate will change in the outermost part of the buffer strip. The authorities instruct delineating the buffer strip for each site so that the moist microclimate and vegetation are preserved [26]—but it is not stated in which area these need to be preserved. On top of the vague Forest Act and confusing instructions, there are no extensive studies or reports on how wide buffer strips have been left in practice. A study of 20 sites found that the average buffer width has been 15 m in Forest Act streamsides [27]. The latest recommendations state that the buffer width should equal the average length of the trees on the sunny side (south and west), but that it can be narrower on the shadowy side (north and east) [26]. In the northern hemisphere, adjacent clear-cuts create the strongest edge effect on southwest-facing edges of forests [28].

In this paper we determine the effects of buffer width and selective logging on the buffer strip on the changes in plant community composition. To accomplish these aims, we designed an experiment where the buffer strip width (15 or 30 m) and selective logging (yes or no) were manipulated and these logging treatments were compared with unmanaged control sites. We report results for vascular plants and mosses that may respond differently to logging [15]. In addition, we test whether southwestern aspect does increase the impact of logging on plant communities, and thus, whether wider buffers are needed on southern and western sides to conserve plant communities efficiently. Here we report the first effects of the treatments after two growing seasons, based on which we discuss the possibilities of reconciliation of commercial forest management and conservation.

## Methods

### Study sites

**Fig. 1:**
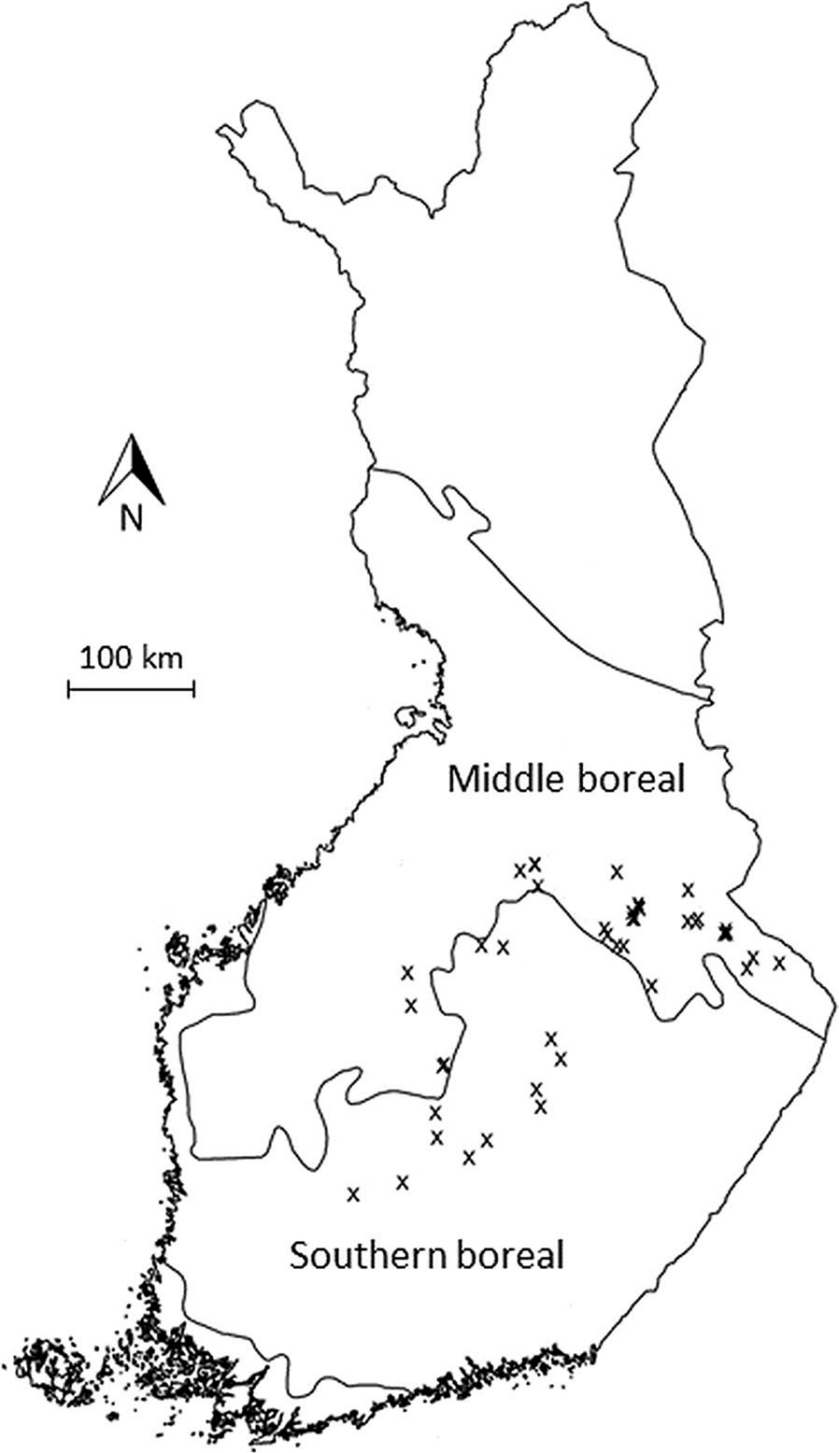
The location of the study sites in Central and Eastern Finland. The sites are located on the southern and middle boreal zones. Map adapted from Maanmittauslaitos, Creative Commons license Attribution 4.0 International

We established 43 study sites in streamside habitats in the southern and middle boreal vegetation zones in Central and Eastern Finland (Fig. 1). Each study site was located on a separate stream from the other sites. All study sites were located in mature managed forests dominated by spruce (*Picea abies* (L.) H. Karst.). Due to the management history, large deciduous trees were mostly absent, but deciduous undergrowth occurred in some sites. The dominant trees were at least 80 years old. All sites had been managed with periodic cover silviculture and were regeneration-ready. The nearest clear-cut was located at least 80 m from the studied site. All water channels were small streams or rivulets with regular, year-round flow. The width of the water channels varied from 0.2 to 4.3 m. The sites were chosen so that there was no extensive regular flooding, but occasional flooding could occur especially near the stream. All of the sites had been classified as Forest Act Habitats by forest authorities. Site-specific information is available in Additional file 1: Table S1.

### Treatments

**Fig. 2:**
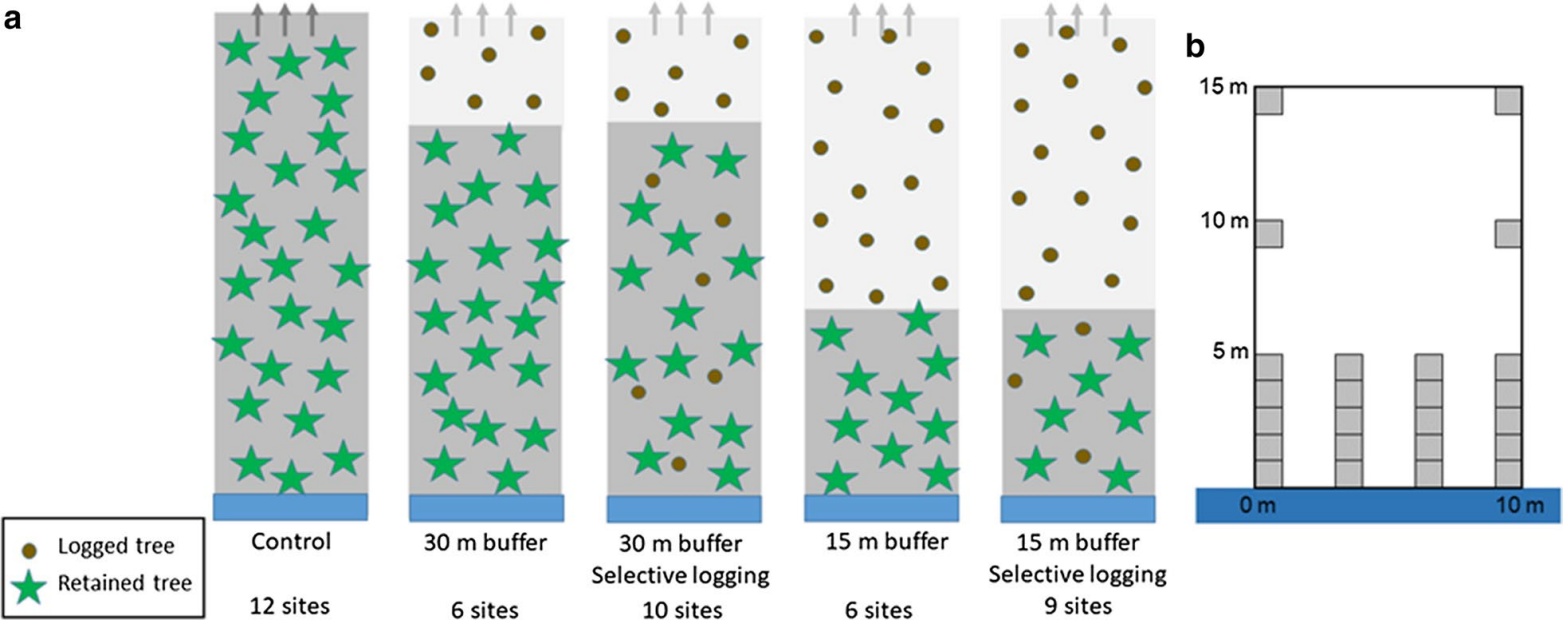
Illustration of the treatments and sampling design. **a** The five types of treatments in the study. Below the name of each treatment is the number of study sites that were included in that type of treatment. The blue bar in the bottom denotes the location of the stream, and no logging was performed on the other side of it. **b** The location of the study plots (grey squares) within the study area (large white rectangle)

During the year of establishment, 2004, all sites were mature forests. Twelve sites were left as controls and no logging was performed in them during the study. In the other 31 sites the upland forest was clear-cut logged during the winter 2005–2006. Between the clear-cut and the stream a forested buffer strip of either 15 or 30 m was excluded from the clear-cut logging. In 12 of these sites no trees were removed from the buffer strip, while in 19 sites the forested buffer strip was selectively logged so that 30% of the basal area of trees was logged, focusing on the largest trees of the stand (i.e. thinning from above). Thus, five different treatments were used in the study sites and they are illustrated in Fig. 2a. The number of sites in each treatment is also provided in Fig. 2a. The treatments were assigned randomly to the sites. In all of the sites mature forest was left standing on the opposite side of the stream, i.e. no logging was performed there.

### Sampling design

On each study site, we established a rectangular 10 m by 15 m study area next to the stream. One of the 10-m sides of the study area followed the stream shoreline. Four sampling lines ran parallel away from the stream, and the starting point of each line was at the stream shoreline (Fig. 2b). Vegetation study plots of 1 m^2^ were placed on each line, but the first plot was not always exactly 1 m^2^, because one of the plot edges followed the meandering shoreline. On each line there were five plots at 0–5 m from the stream (Fig. 2b). On the two outermost lines we placed two additional plots at 10 and 15 m from the stream (Fig. 2b). The sampling was focused near the stream, because the main aim of leaving forested buffer strips is to conserve the species communities at the vicinity of the stream.

In each plot, vascular plants and mosses (Bryophyta) growing on all substrates were identified. The percentage cover of each species was estimated as the proportion of the plot area that the species covered when seen from above. The cover of a species is used as an estimate of its abundance. We collected specimens when needed for microscopic identification of the species and this was done according to the guidelines in Finland. All landowners gave their permission to collect specimens. Identification was mainly done to the level of species, but in some difficult genera the level of genus was used (Additional file 1: Tables S2 and S3). The nomenclature of mosses follows Hodgetts [29] and vascular plants Lampinen and Lahti [30].

The first sampling was carried out before the treatments in the summer 2004 and the second sampling was carried out in the summer 2007, the second growing season after the treatments.

A compass was used to record the aspect of the study area, i.e. the direction of the clear-cut seen from the stream. We transformed the compass point of each site into an index of “southwestern aspect”, which ranges from 0 in northeast to 180 in southwest and gets intermediate values for all compass points between them.

### Statistical analyses

We were interested in the changes that the treatments brought into the community composition of vascular plants and mosses. Bray–Curtis dissimilarity index was used to quantify the changes in the species composition between the pre- and post-logging years on a site. The Bray–Curtis dissimilarity index takes into account the relative abundance of species, and the abundances were square root-transformed prior to the calculation of the dissimilarity in order to reduce the effect of the species that were very abundant.

In control sites, the change in the species composition is due to natural variation between the years 2004 and 2007, and any divergence from this variation in the logged sites is due to the treatments.

The dissimilarity indices were calculated for all sites separately for vascular plants and mosses. Before analysis, the dissimilarities of vascular plant communities were normalized with log_10_-transformation. A simple linear model was built to compare the level of dissimilarities in the four logging treatments to the level of dissimilarities in the control sites.

Regression analysis was used to test for the impact of southwestern aspect on the changes that happen in plant communities after logging with a narrow buffer. Only sites that were logged with 15-m buffer strips were included in this analysis. The index of southwestern aspect was used as the explanatory variable and the Bray–Curtis dissimilarity was the dependent variable. Again, the analysis was done separately for vascular plants and mosses.

To see which species respond to logging, we compared the frequency and relative abundance of each species on the pre- and post-logging years in the sites that were logged with 15-m buffer strips. For this we used the Indicator Species Analysis by Dufrêne and Legendre [31]. The analysis finds species that have high frequency and high relative abundance either before or after logging. We identified species with p < 0.1 as showing an increase or decrease after logging.

All analyses were performed with R version 3.4.0 [32]. Function “vegdist” from package “vegan” [33] was used to calculate the Bray–Curtis dissimilarities. Function “indval” from package “labdsv” [34] was used for the Indicator Species Analysis.

## Results

We found a total of 102 vascular plant species, including one nearly threatened species, *Carex disperma* (Additional file 1: Table S2), and 90 moss species, including the nearly threatened *Plagiothecium latebricola* and the vulnerable *Plagiomnium drummondii* (Additional file 1: Table S3). No invasive alien species were detected.

### Logging treatments

**Fig. 3:**
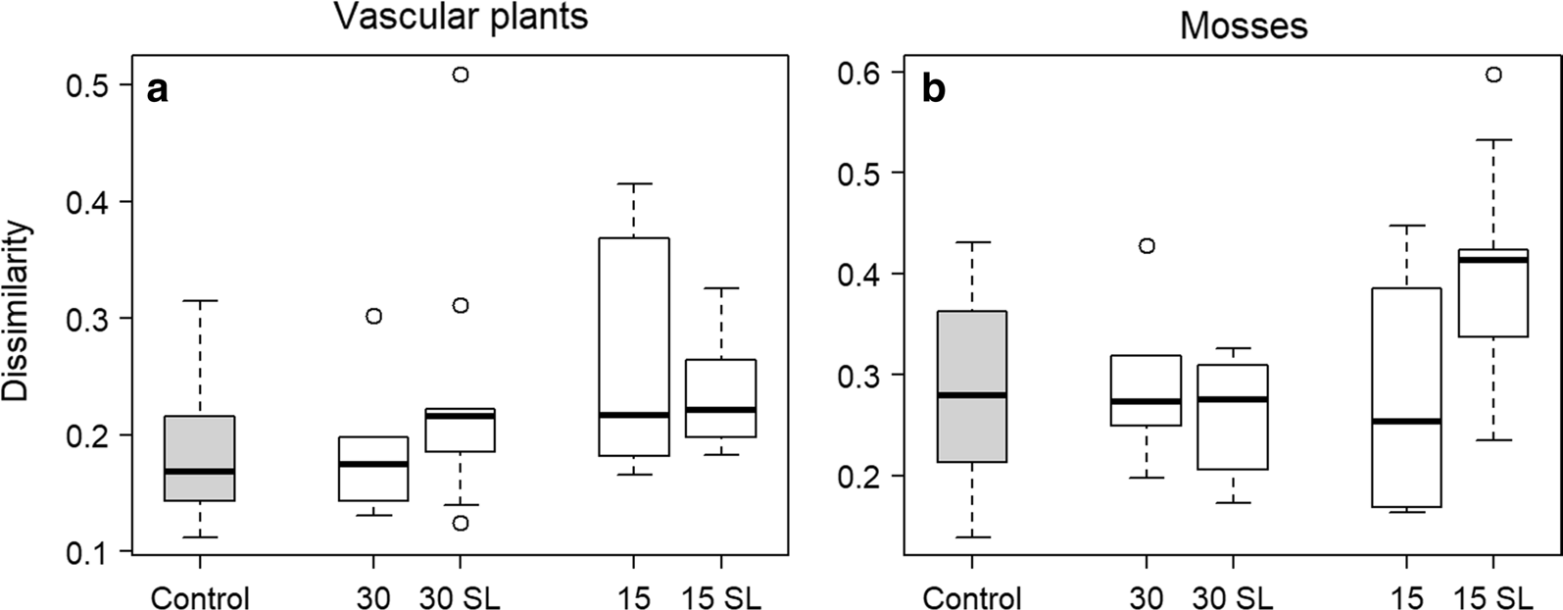
The changes in plant community composition on sites of different treatments. **a** Vascular plants, **b** mosses. The change in the plant community composition on a site is the Bray–Curtis dissimilarity between pre- and post-logging years. The five treatments are: control (no logging), 30-m buffer strip with or without selective logging (SL), and 15-m buffer strips with or without selective logging

Vascular plant community composition changed significantly more on the 15-m buffer strips without selective logging than on controls, and nearly significantly more on the 15-m buffer strips with selective logging than on controls (Table 1, Fig. 3a). On the 30-m wide buffer strips (with or without selective logging) there was no more change than on the control sites (Table 1, Fig. 3a).

**Table 1:** The impact of the four logging treatments on the community change in relation to control sites

	R^2^	Estimate	SE	t	p
*Vascular plants*	0.17				
Intercept (control)		− 0.76			
30 m		0.01	0.07	0.21	0.833
30 m selective logging		0.10	0.06	1.62	0.113
15 m		0.15	0.07	2.15	0.038*
15 m selective logging		0.12	0.06	2.00	0.053
*Mosses*	0.24				
Intercept (control)		0.29			
30 m		0.00	0.05	0.09	0.926
30 m selective logging		− 0.02	0.04	− 0.56	0.577
15 m		− 0.01	0.05	− 0.15	0.884
15 m selective logging		0.11	0.04	2.71	0.010*

The change in the plant community composition on a site is the Bray–Curtis dissimilarity between pre- and post-logging years. The dissimilarity values of vascular plants were log_10_-transformed

Moss community composition changed more on the 15-m buffer strips with selective logging than on controls, while the other three logging treatments did not result in any more change than what was observed on the control sites (Table 1, Fig. 3b).

### Southwestern aspect

**Fig. 4:**
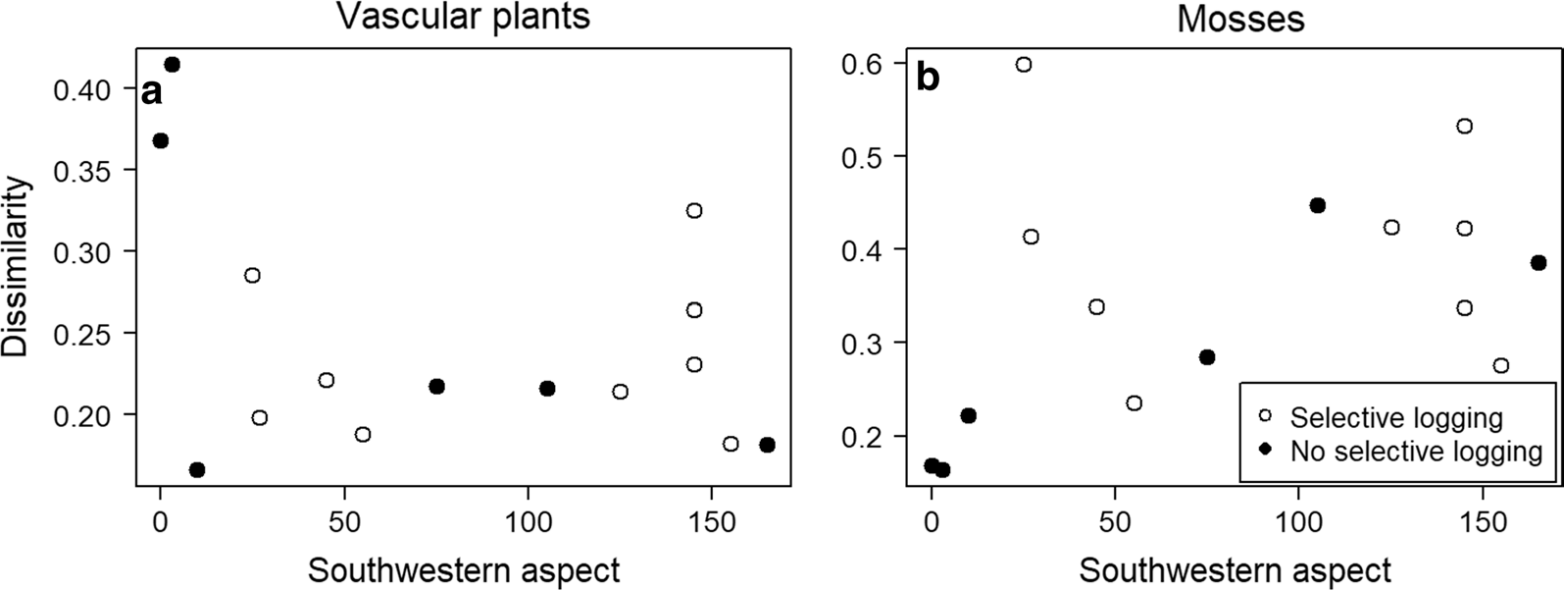
The impact of southwestern aspect on the changes in the plant community composition. **a** Vascular plants, **b** mosses. Southwestern aspect denotes how much the clear-cut is directed towards southwest. Only sites that were logged with 15-m buffer strips are included

The degree of southwestern aspect did not explain the changes that happened in the community composition of vascular plants (regression analysis: F_1,13_ = 1.22, p = 0.29). The largest community changes were in fact observed on two non-selectively logged sites with northeastern aspects (Fig. 4a).

Moss community change was not affected by southwestern aspect, either (regression analysis: F_1,13_ = 2.39, p = 0.15). The site with the largest community change was a selectively logged site with an eastern aspect, while the sites with the smallest community changes were non-selectively logged sites with northeastern aspects (Fig. 4b).

### Responses of individual species

None of the individual vascular plant species showed significant changes in their frequency (number of sites where they occurred) and/or abundance (cover) between the pre- and post-logging surveys. Two species had a nearly significant change: *Carex loliacea* decreased after logging, and *Chamaenerion angustifolium* increased (Table 2).

**Table 2:** Changes in the frequency and abundance of individual species

Group	Species	IV	p
*Vascular plants*			
Decrease after logging	*Carex loliacea* L.	0.27	0.092
Increase after logging	*Chamaenerion angustifolium* (L.) Scop.	0.53	0.088
*Mosses*			
Decrease after logging	*Hylocomium splendens* (Hedw.) Schimp.	0.63	0.096
	*Plagiothecium laetum* Schimp.	0.68	0.052
	*Pleurozium schreberi* (Willd. ex Brid.) Mitt.	0.68	0.021*
	*Sphagnum angustifolium* (C.E.O.Jensen ex Russow) C.E.O.Jensen	0.53	0.004*
	*Sphagnum wulfianum* Girg.	0.34	0.061
Increase after logging	*Plagiomnium cuspidatum* (Hedw.) T.J.Kop	0.27	0.098
	*Plagiothecium denticulatum* (Hedw.) Schimp. var. *denticulatum*	0.57	0.031*
	*Sciuro*-*hypnum curtum* (Lindb.) Ignatov	0.79	0.004*

Results from Indicator Species Analysis comparing the frequency and abundance of each species before and after logging. IV is the indicator value of the species, p is the probability of finding the indicator value. Results are shown for species with p < 0.1, and species with p < 0.05 are marked with *

Among mosses, eight species showed significant or nearly significant changes. *Hylocomium splendens*, *Plagiothecium laetum*, *Pleurozium schreberi*, *Sphagnum angustifolium* and *Sphagnum wulfianum* decreased, while *Plagiomnium cuspidatum*, *Plagiothecium denticulatum* and *Sciuro*-*hypnum curtum* increased (Table 2).

## Discussion

### Logging treatments

We found that irrespective of the selective logging on the buffer strip, 15-m buffers are not enough to protect the vascular plant communities from changes caused by the adjacent clear-cut. For moss communities 15-m buffers were not enough if they were selectively logged. Earlier studies on 10-m wide buffers found significant changes in both vascular plant communities [16] and bryophyte communities [11]. Our results show that to avoid changes in plant communities, the buffer strip should reach more than 15 m from the stream, and that selective logging must not be allowed on such narrow buffer strips.

In the sites with 30-m buffer strips, we observed no changes in community composition of vascular plants or mosses that would be greater than the natural variation. Our result is in accordance with Elliott and Vose [16] who did not observe significant changes in the herbaceous plant community composition in a site logged with 30-m buffer. However, an earlier study of vascular plants and mosses indicated that buffers of 45 m are needed to safeguard their community composition [12], showing that in some cases 30-m buffers may not be sufficient.

The lack of impact of selective logging in 30-m buffer strips suggests that selective logging may not cause large short term changes in the microclimate and growing conditions in the WKH, which appears to be in accordance with the aims of the Forest Act [35]. Interestingly, selective logging at a similar removal level has been found to impact plant communities in Finnish upland spruce forests [3]. The effect of selective logging may be smaller in streamside forests than upland forests because the stream itself may buffer against microclimatic changes [15]. Riparian forests have higher soil and air moisture and smaller temperature variation than upland forests [13, 36]. However, in our study only 30% of the tree basal area was removed and conclusions cannot be done for heavier logging. Zenner et al. [21] studied 46-m wide riparian buffer strips with adjacent clear-cuts, and found that an increasing level of selective logging (from 0 to 30% or 65% removed) resulted in the increasing biomass of understory shrubs, saplings and herbaceous plants.

Our results should be applied cautiously for three reasons. First, our study was very short-term and thus the results apply only for those changes that happened 2 years after logging. The communities changed a lot also in control sites, suggesting that there were also other factors (such as weather conditions) affecting the communities, which may impact the chances of observing logging-related changes. Delayed changes and extinctions may happen [37]. It is also possible that stormy winds result in abundant windfalls [38], posing additional stress on the riparian communities. Second, other species groups may show different responses. For example, liverworts are more sensitive to logging than mosses, and red-listed species of both of these bryophyte groups are especially sensitive [11], and thus their conservation may require wider buffer strips. In addition, although cautious selective logging may not affect ground-dwelling organisms, it will likely affect species that are dependent on the trees themselves, such as epixylic species [39]. Third, wider and non-selectively logged buffers are probably needed on sites with exceptional habitat values, such as places where groundwater is discharged [40], and sites with abundant boulders and/or decaying wood [11]. We conclude that further studies are needed on long-term impacts, sensitive species groups and efficient site-specific planning of buffer strips that avoid changes in the species composition while minimizing economic loss.

### Southwestern aspect

We did not find evidence for the effect of southwestern aspect on the community change of either vascular plants or mosses. For both species groups, the largest changes occurred in fact in sites where the clear-cut was towards north or east. This suggests that in some sites wide buffer strips are required even on eastern or northern sides.

The lack of impact of the aspect in our data is in contrast with earlier studies on the impact of aspect on edge effect [28], and with the recommendation of leaving wider buffers on southern and western sides of streams [26]. The magnitude of community change is probably affected by other local properties than aspect. For example, the extent of moist soil affects the composition of species that are present, but it may also buffer against microclimatic changes after logging. Thus, it is clear that each site should be considered case-by-case to determine the buffer width that is needed to conserve the moist habitat conditions and microclimate.

### Responses of individual species

In our study, none of the vascular plant species showed a significant change in their frequency and abundance after logging, indicating that the changes observed in the community composition of 15-m wide buffers were due to smaller changes in several species. Some species, such as *Carex loliacea*, suffer from the loss of shady, moist conditions or the increase of competition. Others, such as *Chamaenerion angustifolium*, can increase due to increased light, decreased competition or the appearance of bare soil patches. Such increases are well known to occur in boreal forests after clear-cutting [41]. However, the moist riparian habitat together with the buffer strip seems to decrease the amount of changes that happen in the abundance of individual species.

In contrast, several moss species tended to decrease after logging. Such negative tendencies of the mire species *Sphagnum angustifolium* and *S. wulfianum* as well as the abundant forest floor species *Hylocomium splendens* and *Pleurozium schreberi* indicate large changes in the moss layer. Similar declines have been observed in upland forests [3, 15]. The increase of some moss species, such as the generalist forest species *Sciuro*-*hypnum curtum*, could be due to decreased competition, increased light availability or increased leaf litter, but more studies are needed to define the mechanism behind their increase. Interestingly, *Plagiothecium laetum* tended to decrease after logging, while the congeneric *P. denticulatum* increased, indicating differences in their requirements of light and/or moisture. Both of these *Plagiothecium* species grow commonly on convex substrates such as rocks, logs and tree bases [42]. Hylander et al. [11] found that species growing on convex substrates decrease due to logging, while species of concave surfaces are unaffected. Our results do not fully support their findings, because in our dataset *Sphagnum angustifolium* and *S. wulfianum* of concave substrates decreased, while *Plagiomnium cuspidatum*, *Plagiothecium denticulatum* and *Sciuro*-*hypnum curtum* of convex substrates increased. However, the decreasing tendencies of the convex-surface species *Hylocomium splendens*, *Plagiothecium laetum* and *Pleurozium schreberi* do follow the pattern found by Hylander et al. [11].

The changes that happen in species abundances during the 1st years after logging are mostly due to changes in microclimate, possible direct damage by logging machinery and the invasion of pioneer species [11, 15, 43]. Further changes are likely to follow later due to delayed extinctions and colonizations, competition or herbivory [37, 43, 44]. Extinctions are troublesome because many species may not be able to recolonize due to the changed microclimatic conditions or the lack of dispersal propagules. Species that are dependent on ancient forests tend to have poor dispersal abilities [45], and in riparian areas the seed bank lacks many of the species that were present in the standing vegetation [46]. Many species disperse via the riparian corridor, and thus the intactness of the stream hydrology and the riparian forest should be guaranteed at the landscape-level to enable recolonization at disturbed sites [14, 47].

## Conclusions

Buffer strips of 15 m are not sufficient to protect riparian plant communities even in the short term and wider buffers should be used. Selective logging should not be allowed in narrow buffers of only 15 m. On the other hand, buffer strips of 30 m seem to protect community composition at least with the short time period studied here. In practice, it would be essential to delimit the buffer strip width case-by-case based on the extent of the riparian area that needs to be protected. Equally wide buffer strips should be left on both sides of a stream, irrespective of the aspect.

At the current rate of species loss [48, 49], it is important to preserve woodland key habitats by all available means. Buffer strips of at least 30 m are likely needed around other kinds of riparian and moist WKHs as well. Too narrow buffer strips can be considered as a violation of the Finnish Forest Act, which states that the characteristic features of the habitat, such as the microclimate, must not be changed [35].

To mitigate the economic loss of wide buffer strips, cautious selective logging by picking individual trees from within the buffer may be accepted in buffers that are at least 30 m wide. It may be better for the riparian biodiversity to increase buffer width and to allow selective logging on the buffer than to leave only a narrow buffer.

## Additional file

**Additional file 1: Table S1.** Site information. **Table S2.** Vascular plant species found in the study. **Table S3.** Moss species found in the study.

## Abbreviations

WKH: woodland key habitat
SL: selective logging
