# Supplementary material for: The effect of buffer strip width and selective logging on streamside plant communities
Source: BMC Ecol. 2019 Feb 9;19:9. doi: 10.1186/s12898-019-0225-0 (PMC6368960; doi:10.1186/s12898-019-0225-0)
Supplement: Supplementary file 1 — Additional file 1: Table S1. Site information. Table S2. Vascular plant species found in the study. Table S3. Moss species found in the study. [file 12898_2019_225_MOESM1_ESM.pdf]

Additional file 1

For article: The effect of buffer strip width and selective logging on streamside plant communities

Authors: Anna Oldén, Ville A. O. Selonen, Emmi Lehkonen & Janne S. Kotiaho

Table 1. Site information.

Table 2. Vascular plant species found in the study.

Table 3. Moss species found in the study.

Table 1. Information on the study sites: Municipality and name of the location, North and East coordinates in decimal degrees, width of the stream, and the logging treatment: buffer width and selective logging.

Site ID	Municipality	Name	N	E	Stream width (m)	Buffer width	Selective logging
1	Vieremä	Kellopuro	63.83188	26.94863	0.3	15	No
2	Pieksämäki	Koukunjoki	62.39258	26.93276	1.5	15	No
3	Vieremä	Puolinpuro1	63.98682	26.90886	0.5	15	Yes
4	Vieremä	Puolinpuro2	63.98945	26.89380	0.25	30	No
6	Vieremä	Kurkipuro	63.94052	26.66638	0.95	C	No
7	Suonenjoki	Haukipuro	62.60683	27.30495	4.25	30	Yes
8	Pielavesi	Leppipuro	63.39579	26.39757	0.2	15	Yes
15	Kaavi	Kalalamminpuro	63.11614	28.73192	0.55	30	Yes
16	Lieksa	Pieni Ruosmanjärvi 1 SE	63.46902	29.89890	0.45	30	No
17	Lieksa	Pieni Ruosmanjärvi 2 SE	63.46600	29.89691	1.55	15	Yes
18	Lieksa	Tetrikangas NW	63.46808	29.94605	0.3	30	Yes
19	Lieksa	Huosiopuro	63.49229	29.93862	0.65	C	No
20	Lieksa	Hanhilamminpuro	63.28729	30.34200	1.1	15	Yes
21	Lieksa	Palkinoja	63.23884	30.75467	1.6	C	No
22	Lieksa	Lavapuro	63.21131	30.22918	0.35	15	Yes
23	Äänekoski	Kivipuro	62.56329	25.51531	1.2	30	Yes
24	Pihtipudas	Valkeispuro	63.41049	26.05685	0.75	15	Yes
25	Kivijärvi	Kangaspuro	63.20412	24.90234	1.9	30	No
26	Korpilahti	Karhuoja	62.04014	25.42641	1.1	30	Yes
27	Leivonmäki	Väärämäki S	61.90145	25.92199	1.5	C	No
28	Leivonmäki	Hakosjärvi W	62.02793	26.18217	0.55	C	No
29	Korpilahti	Tuppela NE	62.21604	25.39608	0.8	15	No
31	Kuhmoinen	Nokiniemi SW	61.71589	24.93035	0.4	C	No
32	Orivesi	Jokisalo S	61.61620	24.20887	0.6	15	No
33	Karstula	Kurjuksenkulkku NE	62.97202	24.97654	1.65	30	Yes
34	Uurainen	Kivipuro	62.54641	25.48799	2.5	30	No
35	Sotkamo	Ruunapuro	63.93125	28.22158	3.2	C	No

Site ID	Municipality	Name	N	E	Stream width (m)	Buffer width	Selective logging
37	Rautavaara	Risupuro	63.40130	28.30241	1.2	15	No
38	Rautavaara	Nurmespuro	63.40632	28.20288	0.65	15	No
39	Rautavaara	Riitapuro	63.67432	28.56051	0.4	30	Yes
40	Rautavaara	Pieni Sammakkomäki S	63.66626	28.57471	0.2	30	No
41	Rautavaara	Mäntykangas N	63.69888	28.54942	1.05	C	No
42	Nurmes	Niinimäki E	63.56566	29.33364	0.55	30	Yes
43	Nurmes	Rajapuro	63.57713	29.50002	0.3	30	Yes
44	Nurmes	Venepuro	63.55592	29.45544	0.4	C	No
45	Rautavaara	Rinnepuro	63.59531	28.48888	2.05	C	No
47	Rautavaara	Pankapuro	63.63822	28.44861	0.75	C	No
48	Rautavaara	Ukonpuro	63.59369	28.45654	0.7	15	Yes
49	Nurmes	Kuomavaara N	63.78579	29.35355	0.7	15	Yes
50	Varpaisjärvi	Muuraispuro	63.52853	28.01496	0.4	30	No
51	Varpaisjärvi	Juudinsalo S	63.48378	28.04813	0.55	30	Yes
53	Karttula	Suojärvenpuro	62.74879	27.15719	0.95	C	No
56	Pieksämäki	Hietisenpuro	62.26919	26.99563	2.15	15	Yes

Table 2. Vascular plant species found in the study. The IUCN status in Finland is shown if it is other than LC (least concern): NT is nearly threatened. The number of sites where the species was observed is given (total number of sites was 43).

Species	Authors	Status	Sites
Achillea ptarmica	L.		2
Aegopodium podagraria	L.		1
Agrostis canina	L.		12
Agrostis capillaris	L.		3
Agrostis stolonifera	L.		2
Angelica sylvestris	L.		6
Anthriscus sylvestris	(L.) Hoffm.		1
Athyrium filix-femina	(L.) Roth		21
Avenella flexuosa	(L.) Drejer		34
Calamagrostis arundinacea	(L.) Roth		9
Calamagrostis canescens	(Weber) Roth		8
Calamagrostis epigejos	(L.) Roth		1
Calamagrostis neglecta	(Ehrh.) G. Gaertn., B. Mey. & Scherb.		6
Calamagrostis phragmitoides	Hartm.		29
Calla palustris	L.		4
Caltha palustris	L.		2
Carex acuta	L.		1
Carex canescens	L.		37
Carex digitata	L.		21
Carex disperma	Dewey	NT	9
Carex echinata	Murray		4
Carex globularis	L.		27
Carex lasiocarpa	Ehrh.		1
Carex loliacea	L.		8
Carex nigra	(L.) Reichard		9

Species	Authors	Status	Sites
Carex pallescens	L.		1
Carex vaginata	Tausch		7
Chamaedaphne calyculata	(L.) Moench		1
Chamaenerion angustifolium	(L.) Scop.		23
Cirsium heterophyllum	(L.) Hill		1
Cirsium palustre	(L.) Scop.		2
Comarum palustre	L.		9
Convallaria majalis	L.		3
Cornus suecica	L.		3
Crepis paludosa	(L.) Moench		5
Deschampsia cespitosa	(L.) P. Beauv.		23
Dryopteris carthusiana	(Vill.) H. P. Fuchs		33
Dryopteris expansa	(C. Presl) Fraser-Jenk. & Jermy		8
Elymus caninus	(L.) L.		1
Epilobium palustre	L.		2
Equisetum arvense	L.		1
Equisetum palustre	L.		7
Equisetum pratense	Ehrh.		2
Equisetum sylvaticum	L.		37
Eriophorum vaginatum	L.		1
Filipendula ulmaria	(L.) Maxim.		7
Fragaria vesca	L.		2
Galeopsis bifida	Boenn.		3
Galium palustre	L.		15
Galium triflorum	Michx.		1
Geranium sylvaticum	L.		4
Geum rivale	L.		3
Goodyera repens	(L.) R. Br.		4
Gymnocarpium dryopteris	(L.) Newman		39
Hieracium vulgatum	Fr.		2
Juncus filiformis	L.		4
Linnaea borealis	L.		42
Luzula pilosa	(L.) Willd.		27
Lysimachia europaea	(L.) U. Manns & Anderb.		43
Lysimachia thyrsiflora	L.		19
Lysimachia vulgaris	L.		1
Maianthemum bifolium	(L.) F. W. Schmidt		41
Matteuccia struthiopteris	(L.) Tod.		2
Melampyrum pratense	L.		1
Melampyrum sylvaticum	L.		23
Melica nutans	L.		15
Molinia caerulea	(L.) Moench		2
Orthilia secunda	(L.) House		31
Oxalis acetosella	L.		24
Paris quadrifolia	L.		7
Peucedanum palustre	(L.) Moench		4
Phegopteris connectilis	(Michx.) Watt		37
Platanthera bifolia	(L.) Rich.		2
Poa pratensis	L.		1

Species	Authors	Status	Sites
Polypodium vulgare	L.		1
Potentilla erecta	(L.) Raeusch.		1
Prunella vulgaris	L.		1
Pyrola minor	L.		5
Pyrola rotundifolia	L.		2
Ranunculus repens	L.		5
Rubus arcticus	L.		3
Rubus chamaemorus	L.		13
Rubus idaeus	L.		10
Rubus saxatilis	L.		24
Rumex acetosa	L.		2
Scirpus sylvaticus	L.		2
Scutellaria galericulata	L.		3
Silene dioica	(L.) Clairv.		1
Solidago virgaurea	L.		23
Sparganium sp.	L.		1
Spinulum annotinum	(L.) A. Haines		30
Stellaria graminea	L.		1
Stellaria palustris	Ehrh. ex Hoffm.		4
Vaccinium myrtillus	L.		42
Vaccinium uliginosum	L.		1
Vaccinium vitis-idaea	L.		43
Veronica chamaedrys	L.		2
Veronica officinalis	L.		3
Viola ×fennica	F. Nyl.		1
Viola epipsila	Ledeb.		12
Viola palustris	L.		24
Viola riviniana	Rchb.		1

Table 3. Moss species found in the study. The IUCN status in Finland is shown if it is other than LC (least concern): NT is nearly threatened and VU is vulnerable. The number of sites where the species was observed is given (total number of sites was 43).

Species	Authors	Status	Sites
Amblystegium sp.	Schimp.		2
Atrichum sp.	P. Beauv.		17
Aulacomnium androgynum	(Hedw.) Schwägr.		5
Aulacomnium palustre	(Hedw.) Schwägr.		34
Brachytheciastrum velutinum	(Hedw.) Ignatov & Huttunen		4
Brachythecium albicans	(Hedw.) Schimp.		1
Brachythecium salebrosum	(Hoffm. ex F.Weber & D.Mohr) Schimp.		28
Bryum sp.	Hedw.		6
Calliergon cordifolium	(Hedw.) Kindb.		27
Calliergon giganteum	(Schimp.) Kindb.		1
Campylium protensum	(Brid.) Kindb.		1
Ceratodon purpureus	(Hedw.) Brid.		1
Cinclidium stygium	Sw.		4
Cirriphyllum piliferum	(Hedw.) Grout		5

Species	Authors	Status	Sites
Climacium dendroides	(Hedw.) F.Weber & D.Mohr		2
Dichelyma falcatum	(Hedw.) Myrin		1
Dicranella sp.	(Müll. Hal.) Schimp.		5
Dicranum drummondii	Müll.Hal.		1
Dicranum flagellare	Hedw.		3
Dicranum flexicaule	Brid.		1
Dicranum fuscescens	Sm.		37
Dicranum majus	Sm.		43
Dicranum montanum	Hedw.		11
Dicranum polysetum	Sw. ex anon.		40
Dicranum scoparium	Hedw.		40
Ditrichum sp.	Timm ex Hampe		1
Drepanocladus sp.	(Müll. Hal.) G. Roth		4
Fontinalis dalecarlica	Schimp.		1
Hylocomiastrum umbratum	(Hedw.) M.Fleisch.		6
Hylocomium splendens	(Hedw.) Schimp.		43
Hypnum sp.	Hedw.		3
Isopterygiopsis pulchella	(Hedw.) Z.Iwats.		1
Leptobryum pyriforme	(Hedw.) Wilson		1
Mnium hornum	Hedw.		3
Mnium marginatum	(Dicks.) P.Beauv.		1
Mnium stellare	Hedw.		1
Paraleucobryum longifolium	(Hedw.) Loeske		5
Philonotis fontana	(Hedw.) Brid.		1
Plagiomnium cuspidatum	(Hedw.) T.J.Kop.		15
Plagiomnium drummondii	(Bruch & Schimp.) T.J.Kop.	VU	1
Plagiomnium ellipticum	(Brid.) T.J.Kop.		17
Plagiomnium medium	(Bruch & Schimp.) T.J.Kop.		11
Plagiomnium undulatum	(Hedw.) T.J.Kop.		2
Plagiothecium cavifolium	(Brid.) Z.Iwats.		7
Plagiothecium denticulatum var. denticulatum	(Hedw.) Schimp.		26
Plagiothecium denticulatum var. undulatum	R.Ruthe ex Geh.		3
Plagiothecium laetum	Schimp.		43
Plagiothecium latebricola	Schimp.	NT	1
Plagiothecium succulentum	(Wilson) Lindb.		6
Pleurozium schreberi	(Willd. ex Brid.) Mitt.		43
Pogonatum urnigerum	(Hedw.) P.Beauv.		1
Pohlia bulbifera	(Warnst.) Warnst.		2
Pohlia cruda	(Hedw.) Lindb.		3
Pohlia nutans	(Hedw.) Lindb.		29
Pohlia wahlenbergii	(F.Weber & D.Mohr) A.L.Andrews		1
Polytrichastrum sp.	G.L. Sm.		31
Polytrichum commune	Hedw.		40
Polytrichum juniperinum	Hedw.		7
Pseudobryum cinclidioides	(Huebener) T.J.Kop.		37
Pseudotaxiphyllum elegans	(Brid.) Z.Iwats.		1
Ptilium crista-castrensis	(Hedw.) De Not.		14
Racomitrium sp.	Brid.		3
Rhizomnium magnifolium	(Horik.) T.J.Kop.		1

Species	Authors	Status	Sites
Rhizomnium pseudopunctatum	(Bruch & Schimp.) T.J.Kop.		17
Rhizomnium punctatum	(Hedw.) T.J.Kop.		26
Rhodobryum roseum	(Hedw.) Limpr.		19
Rhytidiadelphus subpinnatus	(Lindb.) T.J.Kop.		5
Rhytidiadelphus triquetrus	(Hedw.) Warnst.		19
Sanionia uncinata	(Hedw.) Loeske		37
Sciuro-hypnum curtum	(Lindb.) Ignatov		31
Sciuro-hypnum plumosum	(Hedw.) Ignatov & Huttunen		1
Sciuro-hypnum populeum	(Hedw.) Ignatov & Huttunen		1
Sciuro-hypnum reflexum	(Starke) Ignatov & Huttunen		35
Sciuro-hypnum starkei	(Brid.) Ignatov & Huttunen		40
Sphagnum angustifolium	(C.E.O.Jensen ex Russow) C.E.O.Jensen		23
Sphagnum capillifolium	(Ehrh.) Hedw.		10
Sphagnum centrale	C.E.O.Jensen		11
Sphagnum fallax	(H.Klinggr.) H.Klinggr.		6
Sphagnum girgensohnii	Russow		42
Sphagnum magellanicum	Brid.		3
Sphagnum papillosum	Lindb.		6
Sphagnum quinquefarium	(Braithw.) Warnst.		1
Sphagnum riparium	Ångstr.		9
Sphagnum rubellum	Wilson		1
Sphagnum russowii	Warnst.		9
Sphagnum squarrosum	Crome		27
Sphagnum wulfianum	Girg.		14
Straminergon stramineum	(Dicks. ex Brid.) Hedenäs		11
Tetraphis pellucida	Hedw.		36
Warnstorfia sp.	Loeske		6
